# A randomized controlled trial into the cognitive effects of a computer-tailored physical activity intervention in older adults with chronic disease(s)

**DOI:** 10.1186/s11556-021-00259-9

**Published:** 2021-02-25

**Authors:** Esmee Volders, Renate H. M. de Groot, Juul M. J. Coumans, Catherine A. W. Bolman, Lilian Lechner

**Affiliations:** 1 Faculty of Psychology, Open University of the Netherlands, 6419 AT Heerlen, the Netherlands; 2 Faculty of Educational Sciences, Open University of the Netherlands, 6419 AT Heerlen, the Netherlands; 3 Nutrition and Translational Research in Metabolism (School NUTRIM), Maastricht University, 6200 MD Maastricht, the Netherlands

**Keywords:** Cognition, Physical activity, Ageing, Randomized intervention, Chronic disease, Older adults, Physical activity promotion

## Abstract

**Background:**

Cognitive functioning (CF) is important for wellbeing and an independent life. However, older adults with chronic diseases are at a higher risk of poorer CF levels. Although, research suggests that physical activity (PA) could play an essential role in maintaining good CF, older adults with chronic diseases have low levels of PA. PA interventions to prevent cognitive decline for this specific group exist. Yet, until now these interventions focused on a single specific chronic disease. Active Plus is a proven effective computer-tailored PA stimulating intervention focused on increasing PA in daily life for the older adult population suffering from a broad range of chronic diseases. This study tests the cognitive effects of Active Plus in older adults with chronic diseases.

**Methods:**

In this RCT older adults with at least one chronic disease (≥65 years) were allocated to the intervention group (*N* = 260, mean age = 74.2) or waiting list control group (*N* = 325, mean age = 74.5). In total, intervention group participants received three times computer-tailored PA stimulating advice within four months (i.e., at baseline, after two months, and after three to four months). The online and print delivered advice were tailored to the specific needs and wishes of the participant and focused on incorporating PA in daily life. Baseline and follow-up measurements of the CF verbal memory (Verbal Learning Test), shifting (Trailmaking Test), inhibition (Stop-signal Task) and processing speed (Letter Digit Substitution Test) were assessed after six and 12 months. Intervention effects were analyzed with multilevel linear mixed-effects models adjusted for the clustered design and confounding variables.

**Results:**

The dropout rate was 19.1% after 6 months and 25.1% after 12 months. Although both conditions improved on all verbal memory outcomes after 6 months, and all CF outcomes except inhibition after 12 months, no intervention effects were found, not even in subgroups (*p* > .05).

**Conclusions:**

To our knowledge this is the first study to test the cognitive effects of a computer-tailored PA stimulating intervention in older adults suffering from a broad range of chronic diseases. The effects of the Active Plus intervention were not strong enough to improve CF or prevent cognitive decline. A blended approach, in which this computer-tailored intervention is combined with a face-to-face PA intervention and / or cognitive training, might be a good suggestion to increase the effects of Active Plus on PA and CF in older adults with chronic diseases.

**Trial registration:**

Netherlands Trial Register NL6005; Date of Registration 03-21-2017; https://www.trialregister.nl/trial/6005

## Background

Good cognitive functioning (CF) is important for wellbeing and an independent life [1]. Yet, aging is associated with a decline in CF [2], and especially older adults with chronic diseases are at a higher risk of poorer CF levels than healthy older adults [3]. Largest declines in CF are seen in the executive functions (working memory, inhibition, shifting) and processing speed [1, 4]. These functions are necessary to learn, understand and perform complex daily actions [2], and therefore important for wellbeing and an independent life [1]. Next to a socially integrated network and cognitive challenging leisure activities, regular physical activity (PA) is the factor with highest potential to slow down the rate of cognitive decline and to prevent dementia [5–7].

In fact, these claims are supported by reviews combining different types of research (e.g., cross-sectional studies, animal studies, intervention studies, etc.) on the protective effect of PA on cognitive decline [8–10]. Both cross-sectional and longitudinal cohort studies consistently show the positive association between PA and CF, suggesting that long-term maintenance of sufficient PA may counteract age-related decline of CF [4, 11]. Moreover, active older adults engaging in PA during the lifespan are at a lower risk for cognitive decline and impairment than inactive older adults [8]. In particular, executive functions seem to be the cognitive functions benefitting most from PA [12, 13]. Furthermore, improvements in processing speed and long-term memory have been demonstrated as well in older adults [14].

However, evidence from intervention studies on the effect of PA on CF in older adults is inconsistent [15]. In fact, meta-analytic reviews of randomized controlled trials (RCT) have reported large variations in effects sizes in cognitive outcomes associated with an increase in PA. Some meta-analyses have found moderate cognitive improvements as a result of PA intervention in older adults [9, 12–14], whereas others observed none to limited improvements for delay of cognitive decline in the older adult population even when interventions were successful in increment of PA behavior [16–18]. Although the Cochrane review by Young et al. [16] did not identify any relationship between PA interventions and CF, they deemed it possible that certain subgroups of older adults, such as those with lower starting levels of fitness, could profit more from PA interventions. This is supported by a meta-analysis and systematic review by Cai et al. [19] on effects of exercise on CF in chronic disease patients. They found a positive overall effect of exercise interventions on CF. However, 22 out of 35 included studies only involved patients with Mild Cognitive Impairment or Alzheimer’s disease. In addition, the remainder of the included studies in this meta-analysis focused their intervention on only one chronic disease (i.e. cancer, heart failure) while most older adults suffer from multiple chronic diseases [20].

Furthermore, older adults with chronic diseases have the lowest levels of PA [21, 22], mostly caused by experienced fatigue and pain [21, 23]. Although a few interventions exist to improve PA behavior in this specific population, they are often site-situated, which is high demanding, more expensive, and mainly focus on exercise [24, 25]. Computer-tailoring interventions are a cost-effective solution to improve PA behavior in older adults [26], and can thus also be so for older adults with chronic diseases.

In this light, the computer-tailored PA stimulating intervention Active Plus was developed and evaluated for people aged over 50 years in 2010 [27]. Active Plus participants receive three personalized PA advice letters (online or print delivered) in four months. Earlier research in the general population of older adults of 50 years or over demonstrated that the Active Plus group self-reported to be 1.5 h per week more active at moderate-to-vigorous intensity after one year compared to the control group [28], even in older adults with impaired mobility [29]. At a later time, the computer-tailored program was fitted to a more elderly (≥65 years) population of single adults who suffered from chronic diseases [30]. This adapted version of Active Plus was effective in increasing PA behavior three months after baseline, but no effects were found after six months [31]. However, this concerned an implementation study without a control group, making it impossible to draw definite conclusions on the PA effects of the adapted Active Plus in older adults with chronic diseases.

In conclusion, to our knowledge, there is a lack of cost-effective and easily accessible PA interventions for an elderly population which suffers from a broad range of one or more chronic diseases, and the effects of PA on CF in this population have not yet been tested. Based on these previous studies, we assumed that the computer-tailored intervention Active Plus might improve PA behavior in older adults with chronic diseases and, as a result, could lead to beneficial effects on CF. Our recent RCT showed that Active Plus was only to a limited extend able to improve self-reported PA behavior in chronically diseased older adults six and 12 months after baseline measurements [32]. We did not find any significant intervention effects in objectively measured PA. In addition, subgroup analyses showed that more vulnerable participants (e.g., with a higher degree of impairment, age, or body mass index) benefitted more from the intervention on especially the lower intensity PA behaviors.

In this paper, our main research goal was to test the cognitive effects of Active Plus in older adults with chronic diseases. Though the intervention was individually tailored, it might have been that not all subgroups of participants responded similarly to the Active Plus intervention, as we found in the paper on PA effects [32]. Therefore, we explored whether the cognitive effects differed for subgroups based on degree of impairment, adhering to the PA guidelines (≥ 150 min of moderate-to-vigorous PA), age, gender, body mass index, educational level, and marital status [33].

## Methods

### Study design, setting and population

This study on the effects of the Active Plus intervention on CF was a clustered two-group RCT with a waiting list control group and measurements at baseline, six and 12 months. Ethical approval for the study was obtained from the Research Ethics Committee of the Open University and the trial is registered in the Dutch Trial Register, protocol number NL6005. The study was conducted following the Declaration of Helsinki. A comprehensive rationale and description of the study protocol is published elsewhere [34].

Two socioeconomic comparable neighborhoods or residential areas from seven municipalities were randomly allocated [35] to either the intervention group or the waiting list control group. Due to the nature of the study, blinding was not possible. Municipalities selected between 250 and 2000 independently living adults aged 65 years or older per neighborhood. Potential participants were invited for study participation by their municipalities with an invitation letter via post containing information about the study and an informed consent which could be returned to the researchers from February 2018 until July 2018.

Inclusion criteria were: 65 years or older, fluent in the Dutch language, and suffering from at least one self-reported chronic disease that affects mobility or other physical problems that may affect mobility.^1^ Participants were excluded if they reported severe cognitive problems or were wheelchair users. Participants had to be able to walk at least 100 m, possibly with the help of a walker or walking stick. All participants provided written informed consent.

#### Procedure

**Fig. 1:**
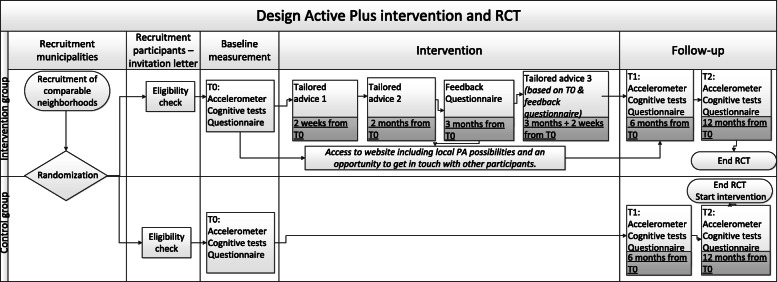
Design of the study

Figure 1 displays the design of the study [34]. To assess PA, participants in both conditions were asked to wear an accelerometer (ActiGraph GT3X-BT) on their right hip for seven consecutive days prior to the CF tests at baseline. The CF tests were conducted by a trained researcher or student at the participants’ home. Inquisit 5 software [36] was used on a tablet (iPad Air 2) to execute the CF tests. To become familiar with the iPad, participants were asked to draw a house and a tree in the Notes application. The CF tests started with the first part of the Verbal Learning Test (VLT), followed by the Trail Making Test (TMT) part A and B, the Stop-signal Task (SST), the Letter Digit Substitution Test (LDST), and the second part of the VLT. Completion time in total was around 45 min. Any occurring disturbances or difficulties in completing the tests were administered to clarify whether the test data were valid. After completing the CF tests, participants received both a paper-based (with a prepaid return envelope) and an online questionnaire (integrated into the project website: www.actief-plus.nl) with the choice to fill out their preferred version (e.g., written or online) within two weeks. In total, 33% chose to fill in this questionnaire online. The questionnaire was used to gather information on demographic variables, but also on concepts that are outside the scope of this manuscript (e.g., self-reported PA, self-reliance, health related quality of life). Thereafter, the four month lasting intervention commenced for the experimental group. Six months (± 3 weeks) and 12 months (± 3 weeks) after the first accelerometer measurement, participants wore that device again, were visited at home for the CF tests and completed a questionnaire following the same procedure as the baseline measurement. After the final assessment (i.e., after 12 months) participants in the waiting list control group received access to the Active Plus intervention.

#### Intervention group

The Active Plus intervention is a computer-tailored intervention aimed at awareness, initiation, and maintenance of PA behavior, especially in daily life, and was originally developed for the general older adult population aged 50 years or over [27]. The content is structured in line with behavioral change theories such as the theory of planned behavior [37], precaution adoption process model [38], integrated model for change (I-Change Model) [39], and self-regulation theory [40]. The intervention was found to be effective in increasing PA behavior in general population of adults over 50 [28]. At a later stage the intervention was adapted with the intervention mapping protocol to an older population of 65 years or older who suffered from chronic diseases [30]. Information was gathered trough literature study, focus groups and expert panels. Although general determinants for PA in older adults with chronic diseases did not seem to differ from the general older adult population, some determinants had a larger influence on PA in this specific population. For example, pain, fear of injury, and lack of energy turned out to be more important barriers. Therefore, the tailored messages were rewritten for the older adults with chronic diseases population to fit more to their needs and requirements. In the present study we tested this adapted intervention. An overview of the adjustments to the adapted intervention made for this study can be found in the design paper [34]. The intervention was tailored to two extra common chronic diseases (neuromuscular and vascular disorders) in addition to the existing 13 common chronic diseases^2^ to which Active Plus was already tailored. Furthermore, information on the risks of sedentary behavior and benefits of PA for CF were extended.

As can be seen in Fig. 1, participants in the intervention group received three times advice, both online on a secured website (if they provided an e-mail address) and on paper (via a letter by mail). The tailored text was the same in both versions, but the online version contained more interactive content (e.g., videos). The first and second personal advice were tailored to the answers that the participants gave in the baseline questionnaire and were received respectively within two weeks and two months after filling in the baseline questionnaire. Three months after the baseline questionnaire, a follow-up questionnaire was conducted that was only used to compose the third advice (i.e. not used to assess any effects). Participants received their third advice within two weeks after completing the follow-up questionnaire. Thus, the intervention period in total lasted four months.

Each advice gave tailored information on PA especially focused on older adults with chronic diseases. Among other things, in the first and second advice participants obtained information on their PA level and whether this was sufficient to gain health effects. Furthermore, they were urged to think of reasons that could motivate them to be sufficiently active and how to overcome barriers in achieving this goal. In addition, suggestions on how to implement PA in daily life and avert fallbacks were given. The third tailored advice provided feedback about the progress in behavior and determinant scores in the previous months. The exact content of all three advice depended on the participants’ characteristics (e.g., age, gender, and presence of chronic disease), psychosocial characteristics/motivational constructs (e.g., awareness, intention, self-efficacy and action planning), their current PA behavior, and to what extent they were willing to alter their behavior. The website and advice also included additional information on local PA possibilities (e.g., walking or cycling routes in their neighborhood or local sports clubs), as well as a user forum, and examples of PA exercises.

#### Waiting list control group

Waiting list control group participants received usual care and had no access to the intervention until the 12-month study period ended. Hereafter they gained access to the Active Plus intervention and received their personalized PA advice on paper and online. But the waiting list control group participants were still visited at home by a researcher or student for the assessment of the CF tests.

### Outcome measures

Table 1 provides an overview of all outcome measures and other assessed variables. The concepts of CF (e.g., verbal memory, shifting, inhibition, processing speed) assessed in this study are chosen because they are known to deteriorate with age and can possibly improve with increased PA behavior (Table 1) [1, 11, 16, 49–51]. There are no normative data available, as these tests, administered with Inquisit 5 software, cannot be compared to pencil and paper versions of the tests [52, 53].

**Table 1:** Outcome measures

Measurement Instrument	Concept	Measure	Scoring/ missing items	Scoring range	Higher score indicates	% valid ^**a**^
***Primary outcome measures***						
VLT	Verbal memory	Learning curve ratio	(Trial 1 + (Trial 2-Trial 1) + (Trial 3-Trial 2) + (Trial 4-Trial 3) + (Trial 5-Trial 4)) / 5	0–3 words per trial	Better learning capacity	98%
		Mean number of recalled words trial 1–5	(Trial 1 + Trial 2 + Trial 3 + Trial 4 + Trial 5) / 5	0–15 words	Better short-term verbal memory	
		Number of words recalled in delayed trial		0–15 words	Better long-term verbal memory	
TMT	Shifting	Time to complete part B minus time to complete A in sec		0-∞ sec	Worse shifting capacity	96%
SST	Inhibition	SSRT in ms	The SSRT is estimated in accordance with De Jong et al. [41] and Tannock et al. [42]. Negative SSRT values are excluded from the analyses [43].	0–1500 ms	Worse inhibition	90%
LDST	Processing speed	Number of correct substitutions		0–125 subs	Better processing speed	96%
***Demographic and health characteristics***						
ActiGraph GT3X-BT	PA	MVPA minutes per week	Data downloaded with frequency extension on [44]. Valid if worn 4 days during 10 h or more [45]. Non-wear definition by algorithm of Choi et al. [46]. PA scoring by Freedson-VM cut-off points [47] and by Aguilar-Fariaz cut-off points [48].	0–6720 min	More MVPA	96%
		LPA minutes per week		0–10,080 min	More LPA	

Abbreviations: *VLT* verbal learning test, *TMT* trail making test, *SST* stop-signal task, *LDST* letter digit substitution test, *PA* physical activity, *SSRT* stop-signal reaction time, *MVPA* moderate-to-vigorous physical activity, *LPA* light physical activity. ^a^ Test outcomes were excluded if scores were deemed invalid by test administer when 1) technical problems occurred, 2) participants refused to complete a test or lacked motivation, 3) participants had physical limitations (arm amputated, hearing loss etc.) or cognitive restrictions (participant is unable to understand the instruction), or 4) participants deviated from the instructions

In the Verbal Learning Test (VLT) [49, 54], which assesses verbal memory, 15 common monosyllabic words representing concrete objects were presented one by one on an iPad screen in fixed order, with a presentation time of one second and an interstimulus interval of one second. Afterwards participants were asked to verbally recall the words they had remembered. The first trial was followed by four more trials in which the words were presented in identical order and each followed by an immediate free recall procedure. After a delay of 15–25 min in which the remaining CF tests were assessed, and unexpectedly for the participants, the instruction was given to recall the 15 words learned once more. Finally, a recognition trial was administered where participants had to recognize the 15 learned words out of 30 words. Outcome measures for the VLT were the learning curve ratio over trials 1–5, the mean number of recalled words in trial 1–5, and the number of words recalled in delayed trial (Table 1).

During the Trail Making Test (TMT) part A and B [55], which can be used to asses shifting, participants had to draw lines with their fingers connecting 25 randomly placed numbers in the correct order (part A) or numbers and letters alternatively (part B). The time in seconds required to complete the task was noted for each task. The outcome measure for the TMT was the time to complete part B minus the time to complete part A.

In the Stop-signal Task (SST) [56], which is an inhibition task, participants had to quickly press the left-hand button if the arrow on the iPad screen pointed to the left and press the right-hand button if the arrow pointed to the right. However, when a signal beep was played after the presentation of the arrow, participants should have inhibited their reaction and withheld from pressing either of the buttons. These beeps occurred in 25% of the trials. Firstly, participants could practice the task in a block of 32 trials. Afterward, three blocks of 64 trials were completed with 10 s of rest in between blocks. The stop-signal delay between presentation of the arrow and signal beep was varied and depended on participants’ performance. The delay, which started at 250 milliseconds (ms), was increased with 50 ms if the previous inhibition was successful. The delay got smaller with 50 ms if the previous inhibition was unsuccessful. This stop-signal delay staircase design ensured that participants were able to inhibit their response on approximately half of all trials. The stop-signal reaction time (SSRT) was the outcome measure.

During the Letter Digit Substitution Test (LDST) [50], which is a processing speed task, participants were presented with a matrix. Odd rows contained letters; even rows contained empty answer boxes. The task was to translate the letters by clicking the corresponding digits with the help of a provided key. After a practice round of 10 letters, the participant had 60 s to replace as many randomized letters with the appropriate digit indicated by the key. The outcome measure for the LDST was the number of correct substitutions made in 60 s.

#### Demographic and health characteristics

As age, gender, educational level, marital status (living together with a spouse or living single), body mass index (BMI), and physical impairment, are known to influence PA behavior [57] and some also CF [58], these factors were assessed at baseline. Educational level is categorized into low (i.e., primary, basic vocational, or lower general school), moderate (i.e., medium vocational school, higher general secondary education, and preparatory academic education), or high (i.e., higher vocational school or university level) according to the Dutch educational system.

BMI is defined as the body mass (in kg) divided by the square of body height (in cm). The degree of physical impairment is measured with a self-report questionnaire [30]. The participant stated for 14 common chronic diseases (e.g., cardiovascular, osteoarthritis) and physical conditions (e.g., hearing or visually impaired) to what degree he/she was limited in his/her PA behavior by one of the diseases mentioned or by another disease not mentioned. For each chronic disease, the participant scored the degree of impairment on a 5-point scale ranging from 0 = not applicable, 1 = not at all/hardly, 2 = a little, 3 = very, to 4 = extremely. Consequently, degree of impairment was computed into three categories following the next rules: (1) Little impaired: a maximum score of one on at least one question, (2) Medium impaired: a maximum score of two on at least one question, (3) Very impaired: at least a score of three or four on at least one question. PA was objectively measured using the ActiGraph GT3X-BT (ActiGraph, Pensacola, FL, USA). The accelerometer was placed on the right hip with an elastic belt. Participants were asked to wear the accelerometer for seven consecutive days. However, during the night participants were not obliged to wear the device. While showering or swimming, the meter had to be removed.

#### Sample size and statistical power

Based on the general effect size in a review by Northey et al. [9] of earlier PA intervention studies to improve CF, we used an estimated effect size (ES) of 0.3. Because of the multilevel design, the sample size had to be inflated. Therefore, based on the intra-cluster correlation (ICC) of previous Active Plus projects (ICC < 0.01) an estimate of ICC of 0.01 was used. Statistical power analysis using G*Power [59] (ES = 0.30; power = 0.80; ICC = 0.01) showed that 190 participants per group were required. We expected a 30% dropout rate at 12 months based on our previous study [28]. Therefore, 270 participants needed to be enrolled at baseline in both the intervention group and the waiting list control group.

#### Statistical analyses

Baseline differences between both groups were tested with a χ^2^ test for categorical variables, a Mann–Whitney U-test for continuous variables with skewed distributions, and an independent two-sample t-test for normally distributed continuous variables. For further analyses, we log transformed the non-normally distributed TMT outcome measure, and for the SST outcome measure SSRT, we applied a square root transformation. To assess predictors of dropout at six and 12 months, logistic regressions with condition, baseline outcome measures, demographics, degree of impairment regarding chronic diseases and amount of moderate-to-vigorous PA and light PA were performed.

Linear mixed-effects models were used to assess intervention effects on CF. With participants originating from different municipalities, it was expected that their data was clustered. Therefore, we applied multilevel analyses with participants nested in municipalities, with level one being the different time points, level two the participant, and level three the municipality. The analyses revealed that the ICC values for all CF outcomes were smaller than 0.01. Consequently, two-level analyses were performed for all outcomes to assess intervention effects. Time, group, and the interaction between time and group were added to the models as fixed effects to assess intervention effects over time. Intervention effects between intervention group and control group were compared between baseline and six months follow-up and between baseline and 12 months follow-up. All models were fitted using the maximum likelihood procedure. For all analyses age, gender, educational level, marital status, BMI, degree of impairment, and objectively measured weekly minutes of moderate-to-vigorous PA and light PA were added as covariates, as all variables except BMI and degree of physical impairment contributed significantly to the multilevel models. In addition, BMI and degree of physical impairment were significant covariates for PA behavior [32]. Continuous variables were standardized. Furthermore, confidence intervals (CI) and ES were calculated for all outcomes. ES were calculated by standardizing all variables [60]. Analyses were conducted on all available and valid data without any ad hoc imputation [61].

For exploratory purposes, differences regarding intervention efficacy were assessed for degree of impairment, age, gender, educational level, marital status, BMI, moderate-to-vigorous PA and light PA. Three-way interaction terms (time × group × covariate) of each significant covariate were separately added to the model. When a three-way interaction term was significant, subgroup effects were examined by repeating the primary analysis. In these multilevel analyses, the two-level data structure was applied again. Subgroups were defined by ‘logical’ cut-off points. For categorical variables the different categories of the covariate were used. For the continuous variable BMI, the groups were split by normal weight (< 25 kg/m^2^), overweight (25–29.9 kg/m^2^) or obese (≥30 kg/m^2^), based on cut-off points used by the WHO. For age, the limit was at 80 years or older. The WHO calls this group the oldest-old [33]. For minutes of moderate-to-vigorous PA the groups were split into meeting the moderate-to-vigorous PA minutes guidelines (≥ 150 min) and not meeting the guidelines. Since interaction terms have less power, the significance levels were set at *p* < 0.10 for the interaction term [61]. Significance levels for all other analyses were set at *p* < 0.05. All analyses were conducted using R [62].

## Results

### Study population

**Fig. 2:**
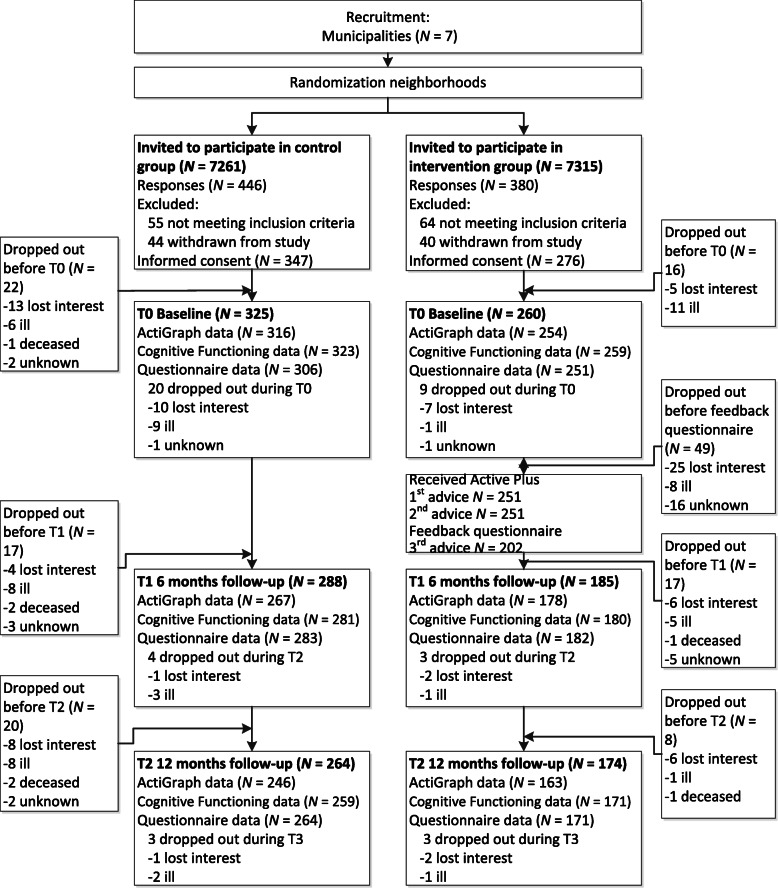
Flow diagram of the study

Of the 623 participants who provided informed consent and were included in the study, 38 withdrew from the study before baseline. As can be seen in the flowchart (Fig. 2), a total of 585 participants provided some baseline data and were therefore included in the analyses (at baseline eight participants provided only one measurement type (ActiGraph, CF tests, or questionnaire), and 23 participants provided only two measurement types). At 6 months 19.1% (112/585) of the participants dropped out and at 12 months this rate was 25.1% (147/585). Dropout at both six and 12 months after baseline was more likely for participants in the intervention group (6 months: OR = 7.61, 95%CI = 3.90;15.96, *p* ≤ 0.001; 12 months: OR = 3.35, 95%CI = 2.01;5.72, *p* ≤ 0.001) and elder participants (6 months: OR = 1.07, 95%CI = 1.02;1.14, *p* = 0.01; 12 months: OR = 1.06, 95%CI = 1.01;1.11, *p* = 0.02). At 12 months participants who had lower minutes of moderate-to-vigorous PA per week at baseline were more likely to drop out (OR = 1.00, 95%CI = 1.00;1.00, *p* = 0.02). It also was recorded why people dropped out of the study as can be seen in the flowchart. Most intervention group participants (48 out of 89 dropouts) dropped out because they lost interest. This occurred mainly during the first part of the intervention when they had to fill in a follow-up questionnaire needed to compose the third advice. In contrast, most control group participants (30 out of 64 dropouts) dropped out of the study because of being too ill to continue.

As can be seen in Table 2, the participants had an average age of 74.3 (±6.4) years; 51.6% was male; 51.2% was low-educated (i.e., primary, basic vocational, or lower general school); 46.1% of the participants was medium physically impaired and 40.5% was very impaired. Osteoarthritis (51.7% of all participants), vascular diseases (44.6%) and heart diseases (37.2%) were the most common chronic diseases participants suffered from. Participants suffered from an average of 3.5 chronic diseases or physical impairments per person. The control and intervention group did not differ on baseline characteristics (Table 2).

**Table 2:** Baseline participant characteristics of the control group and the intervention group

	**Control Group**	**Intervention Group**	***p*-Value**
	**(*N*** **= 325)**	**(*N*** **= 260)**	
Demographic characteristics			
Age in years, mean (SD)	74.5 (6.2)	74.2 (6.6)	0.62
Gender, *N* (%)			0.59
Male	164 (50.5%)	138 (53.1%)	
Female	161 (49.5%)	122 (46.9%)	
Marital status, *N* (%)			0.09
Living single	50 (16.6%)	56 (22.6%)	
Living together	252 (83.4%)	192 (77.4%)	
Education, *N* (%)			0.54
Low	151 (50.3%)	127 (52.3%)	
Moderate	60 (20.0%)	54 (22.2%)	
High	89 (29.7%)	62 (25.5%)	
Health-related characteristics			
BMI, median (IQR)	26.9 (24.1–29.4)	26.9 (24.4–29.8)	0.35
Degree of impairment, *N* (%)			0.39
Little impaired	34 (11.1%)	29 (11.6%)	
Medium impaired	134 (43.8%)	123 (49.0%)	
Very impaired	138 (45.1%)	99 (39.4%)	
MVPA in min/wk., median (IQR)	145 (57.0–289.8)	141.5 (60.5–261.0)	0.70
LPA in min/wk., mean (SD)	2486 (641)	2494 (674)	0.88
CF outcomes			
VLT – learning curve ratio, mean (SD)	1.84 (0.55)	1.81 (0.55)	0.50
VLT – mean no. words recalled trial 1–5, mean (SD)	7.16 (2.11)	7.07 (2.05)	0.62
VLT – no. words delayed recall, mean (SD)	7.42 (3.27)	7.39 (2.97)	0.90
TMT – time B-A in sec, median (IQR)	30.32 (13.97–53.85)	28.45 (13.87–52.40)	0.76
SST – SSRT in ms, median (IQR)	162.75 (111.58–225.25)	166.25 (115.17–226.42)	0.83
LDST – no. correct subs, mean (SD)	10.81 (4.28)	10.85 (4.14)	0.92

Abbreviations: *SD* standard deviation, *IQR* Inter Quartile Distance, *BMI* body mass index, *MVPA* minutes of moderate-to-vigorous physical activity per week, *LPA* minutes of light physical activity per week, *CF* cognitive functioning, *VLT* verbal learning test, *TMT* trail making test, *SST* stop-signal task, *SSRT* stop-signal reaction time, *LDST* letter digit substitution test. non-normally distributed variables tested with the Mann–Whitney U test

Overall, 95.6% of all CF tests were deemed valid (see Table 1). The SST had most occurrences of invalid data both at baseline, and at six and 12 months after baseline. Around 10% of all Stop-Signal Task tests was considered invalid, mostly due to physical limitations or not understanding the instructions (74 out of 149 invalid tests). The first questionnaire was completed online by 33% of the participants. The second and third questionnaire was completed online only by 16% of the participants.

### Intervention and moderation effects

Both groups (i.e., intervention group and waiting list control group) scored significantly better on three out of six CF outcomes after six months (all verbal memory outcomes), and on all outcomes except inhibition after 12 months. However, no significant intervention effects were found (Table 3) both six months after baseline and 12 months after baseline on CF outcomes. Furthermore, the outcomes of the moderation analyses are displayed in Table 4. Although some three-way interactions between group, time and covariates were significant (*p* < 0.1), no significant intervention effects (*p* < 0.05) in subgroups were found.

**Table 3:** Intervention effects (Group × Time interaction) on CF outcomes for 6 and 12 months follow-up adjusted for the covariates*

	***N***	Effect After 6 Months					Effect After 12 Months				
		Coeff.	SE	95% CI	***p***	ES	Coeff.	SE	95% CI	***p***	***ES***
VLT – learning curve ratio	522	0.04	0.04	−0.03;0.11	0.30	0.07	0.07	0.04	−0.01;0.14	0.09	0.11
VLT – mean no. words recalled trial 1–5	522	0.07	0.13	−0.19;0.33	0.60	0.03	0.05	0.13	−0.22;0.31	0.74	0.02
VLT – no. words delayed recall	520	0.09	0.20	−0.31;0.48	0.67	0.03	0.20	0.20	−0.20;0.60	0.33	0.06
TMT – time B-A in sec ^a^	516	−0.02	0.02	−0.06;0.03	0.40	−0.08	0.03	0.02	−0.02;0.07	0.23	0.11
SST – SSRT in ms ^b^	499	0.10	0.44	−0.77;0.96	0.82	0.03	−0.58	0.45	−1.47;0.31	0.20	−0.17
LDST – no. correct subs	520	0.29	0.32	−0.39;0.91	0.36	0.07	0.29	0.33	−0.35;0.93	0.37	0.07

Abbreviations: *SE* standard error, *CI* confidence interval, *ES* effect size, *CF* cognitive functioning, *VLT* verbal learning test, *TMT* trail making test, *SST* stop-signal task, *SSRT* stop-signal reaction time, *LDST* letter digit substitution test. * Effects are reported as intervention group vs control group as the control group served as a reference group. ^a^ TMT – time B-A in sec was log transformed. ^b^ SST – SSRT in ms was square root transformed

**Table 4:** Moderation of intervention effects (Group × Time interaction) on CF outcomes for 6 and 12 months follow-up in subgroups*

	Subgroup	Effect After 6 Months				Effect After 12 Months		
		***N***	Coeff.	95% CI	***p***	Coeff.	95% CI	***p***
VLT – learning curve ratio	< 80 years	412	0.03	−0.05;0.10	0.51	0.06	−0.02;0.14	0.14
	≥80 years	110	0.10	−0.10;0.29	0.33	0.09	−0.11;0.30	0.38
VLT – mean no. words recalled trial 1–5	Meeting MVPA guidelines^1^	258	−0.04	−0.40;0.32	0.84	−0.13	− 0.49;0.23	0.49
	Not meeting MVPA guidelines^1^	264	0.19	−0.17;0.55	0.31	0.24	−0.13;0.62	0.21
LDST – no. correct subs	Low education	269	0.31	−0.52;1.13	0.47	0.48	−0.39;1.35	0.28
	Moderate education	107	−0.36	−1.79;1.07	0.62	−1.37	−2.86;0.13	0.07
	High education	144	0.71	−0.48;1.90	0.24	1.15	−0.04;2.35	0.06

Abbreviations: *CI* confidence interval, *CF* cognitive functioning, *VLT* verbal learning test, *SST* stop-signal task, *SSRT* stop-signal reaction time, *LDST* letter digit substitution test, *MVPA* moderate-to-vigorous physical activity per week.* Effects are reported as intervention group vs control group as the control group served as a reference group in the different subgroups. Subgroups were predetermined based on paper on effects on PA. ^1^ Assessed at baseline; Guidelines prescribe 150 min of MVPA per week

## Discussion

To the best of our knowledge, this is the first study to assess the cognitive effects of a computer-tailored PA intervention in older adults suffering from a broad range of chronic diseases. Additionally, this study explored the effectiveness of the intervention on cognitive functions in relevant subgroups based on demographics, PA behavior, and level of impairment. Although both the intervention group and the control group significantly improved most of their CF test scores over time, there were no effects of the Active Plus intervention on the assessed domains of CF (verbal memory, shifting, inhibition, and processing speed), nor did we find any cognitive effects of the intervention in subgroups of older adults with chronic diseases.

The finding that both groups improved significantly on all verbal memory outcomes after six months, and on all CF outcomes except inhibition after 12 months, can be explained in multiple ways that might have occurred next to each other. First, the increased CF scores for both groups may have been caused by a learning effect. In CF literature, various reasons have been discussed to explain the improved scores originated by practice, such as reduced anxiety or increased familiarity with the testing environment and procedural learning [63]. Because almost all participants had no earlier experience with partaking in a study and because they were not confident what the CF tests were all about, it is plausible that this could have elicited stress. Second, the house visits necessary to conduct the CF tests can have caused an improved CF in both groups, because of the personal attention participants received from the researcher or student, as previous research has suggested the existence of the Hawthorne effect [64]. Third, the assessment of the PA outcome measures themselves could have led to better CF. As both the intervention group and the control group received the same assessments, it is plausible that wearing the accelerometer and filling in the questionnaire (which focused on PA behavior, motivation for PA and intention to PA) led to higher awareness about their own current PA behavior and as a result led to an increase in PA behavior. However, in this RCT, the control group did not improve on either of the eight PA outcomes assessed [32]. Therefore, it is less likely that the PA measurements themselves have caused an improved CF.

However, the most likely explanation for the contradictory findings between earlier studies on the relationship between PA and CF, and this RCT, is the minimal effectiveness found of Active Plus on PA behavior in older adults with chronic diseases. The Active Plus intervention aims to stimulate PA behavior in older adults with chronic diseases and has already been proven to increase PA behavior effectively in the general older adult population [28]. However, in this RCT, the intervention was only able to improve self-reported PA to a limited extent and did not improve objectively measured PA in older adults with chronic diseases [32]. The small significant improvements (walking, cycling, gardening) were found on the PA behaviors performed at a lower intensity than necessary, for example, for sports. Even though there still is a lot unknown about the PA characteristics that lead to optimal results for CF [13], it is possible that the intensity of PA may be important [16], and some research suggests that a moderate-to-vigorous intensity is needed [9, 65]. Therefore, the limited effects we found on self-reported PA may have been too weak to improve CF. The mechanisms that may have led to the poor effects on PA behavior might be due to the target population, the relatively high baseline amounts of MPVA (less room for improvement), the nature of the intervention Active Plus and the design of the RCT itself. However, these explanations are beyond the scope of this paper and have been discussed thoroughly in our paper on the effects of the Active Plus intervention on PA [32].

There are some examples of PA interventions that did have a positive effect on CF tested with an RCT [66–69]. The study by Albinet et al. [66] found an intensive 12 weeks aerobic exercise program effective in increasing executive functioning in older adults as opposed to a stretching program. However, the study had a small sample size (*N* = 24) and used only one CF test. The study by Muscari et al. [67] included 120 healthy older adults. The intervention, which consisted of supervised endurance exercise training three times a week for 12 months, was effective in reducing progression of cognitive decline. This study only assessed the Mini-Mental State Examination as a measure of cognitive function, which is not a reliable test for change scores in this population [70]. Best et al. [68] examined the effects of resistance training once or twice weekly as opposed to twice-weekly balance and tone training. Resistance training was beneficial for executive function and the memory domain. However, this study only included women, which affects the generalizability and had a relative low compliance to the program. All of the above interventions were site-situated and therefore quite expensive to execute and demanding for the participants. Liu-Ambrose et al. [69] tested the effects of a home-based resistance training, balance, and aerobic program on executive functioning. The program significantly improved inhibition in the intervention group versus the control group. However, this study included only participants who had fall incidents and found significant effects on only one of the three executive functioning outcomes (inhibition, updating, set-shifting). Furthermore, this study still relied on the deployment of trained personnel. In conclusion, the studies mentioned above have important limitations, were expensive to execute and demanding for participants. Higher quality studies are needed to clarify the association between PA interventions and cognitive function and to determine which types of PA will have the greatest benefit on specific cognitive domains.

While only the computer-tailored PA stimulating Active Plus intervention is not sufficient to increase PA in the general older adults with chronic diseases population and possibly thereby have an intervention effect on CF, a possible solution to enhance the effect could be a blended approach in which this computer-tailored intervention and face-to-face contact are combined [71]. Especially in a more elderly population that maybe is less internet and more personal contact-oriented. A blended approach could be a cost-effective solution, as it implies less costly face-to-face contact and improved feeling of self-regulation. For example, the Active Plus intervention, which contains solely personalized advice on how to implement PA in daily life, could be combined with face-to-face contact with a physiotherapist or weekly meetings with a PA group for older adults. Especially as older adults are known to prefer to exercise in groups as opposed to exercise alone [72]. A blended approach is increasingly being applied in both healthcare and mental healthcare [71]. There are already some examples of blended approach interventions aimed at promoting PA in older adults [73, 74]. However, only a few studies exist, and results are mixed. To our knowledge, there are no blended interventions to improve CF or prevent a further cognitive decline through improving PA.

Furthermore, to improve effectiveness of Active Plus, the intervention could be enriched with cognitive training to increase intervention effects on CF. There are already some indications that combining both physical training and cognitive training could lead to better outcomes on CF, since cognitive training appears to improve other CF domains than PA in older adults [75, 76], and this results in larger effects on CF in older adults than solely physical or cognitive training [77, 78]. However, not all studies show that cognitive training programs or intellectually demanding activities enhance general CF. At best, they find that such interventions boost one’s performance in tasks similar to the trained task, and do not extend to other cognitive domains. This is an important issue for future research [79].

Some strengths of the study should be mentioned. First, the current study has a strong research design, as RCT’s are considered the golden standard in effectiveness studies. Second, our study participants were quite mixed and generalizable to a general older adults with chronic diseases population or even the general older adult population. As an example, our research population had almost equal numbers of male and female participants, and the majority of the participants was low educated (e.g., 51%). In addition, our research sample had BMI levels [80] and a mean number of comorbidities (3.5) [81] equal to the general older adult population in the Netherlands. However, selective response is probable. Despite these strengths, the study also had some limitations. Although the CF tests are well-validated sensitive tests, sensitivity still might be too low. However, this is an issue in every study that measures CF, and computer-based tasks (e.g. this RCT assessed CF on an iPad) are actually suggested to improve sensitivity of cognitive assessments [82]. Furthermore, the selective dropout (i.e., older participants, during the intervention period, and those with lower baseline levels of moderate-to-vigorous PA) may have influenced our results, although this is expected to be less harmful because of the reasonably low dropout. In (partly) digital health interventions a dropout rate of 25.1% per cent is thought of as low [83]. Furthermore, we accounted for these possible confounding variables.

## Conclusions

To our knowledge this is the first study to test the cognitive effects of a computer-tailored PA stimulating intervention in older adults suffering from a broad range of chronic diseases. Our results indicated that both conditions improved on all verbal memory outcomes after six months, and all CF outcomes except inhibition after 12 months. However, the Active Plus intervention was not more effective than the waiting list control group in improving cognitive functioning six and 12 months after baseline measurements. As the Active Plus intervention was not effective enough on its own to increase PA behavior and thereby improve CF or prevent cognitive decline through increased PA, these limited effects are understandable. A blended approach, in which this computer-tailored intervention is combined with a face-to-face PA intervention and / or cognitive training, might be a good suggestion to improve the effects of Active Plus on PA and CF in older adults with chronic disease(s). Future studies could fruitfully explore this opportunity further.

## Data Availability

Study data are available from the corresponding author on reasonable request.

## Appendix

### I Raw outcomes

**Table 5:** Raw CF outcomes at baseline and follow-up measurements by treatment group*

	Measurement	Control Group		Intervention Group	
		***N***	Mean (SD)	***N***	Mean (SD)
VLT – learning curve ratio	Baseline	316	1.84 (0.55)	254	1.81 (0.55)
	6 months	278	2.04 (0.56)	174	2.04 (0.56)
	12 months	254	2.09 (0.57)	168	2.13 (0.55)
VLT – mean no. words recalled trial 1–5	Baseline	316	7.16 (2.11)	254	7.07 (2.05)
	6 months	278	8.16 (2.20)	174	8.17 (2.25)
	12 months	254	8.72 (2.48)	168	8.67 (2.25)
VLT – no. words delayed recall	Baseline	312	7.42 (3.27)	254	7.39 (2.97)
	6 months	275	8.85 (3.17)	178	8.88 (3.23)
	12 months	254	9.15 (3.51)	170	9.27 (3.26)
TMT – time B-A in sec	Baseline	300	42.24 (48.77)	249	39.40 (39.93)
	6 months	272	36.41 (38.97)	176	30.12 (32.19)
	12 months	251	31.81 (32.11)	167	33.32 (34.17)
SST – SSRT in ms	Baseline	281	177.53 (99.70)	221	177.31 (95.88)
	6 months	245	163.37 (84.74)	156	165.37 (77.39)
	12 months	226	161.50 (76.54)	139	146.90 (74.33)
LDST – no. correct subs	Baseline	303	10.81 (4.28)	249	10.85 (4.14)
	6 months	277	11.38 (4.08)	172	12.07 (3.83)
	12 months	249	11.64 (4.39)	158	12.08 (4.23)

Abbreviations: *SD* standard deviation, *CF* cognitive functioning, VLT, verbal learning test, TMT, trail making test; SST, stop-signal task, *SSRT* stop-signal reaction time, *LDST* letter digit substitution test. * Summary statistics using all the available and valid individual data at baseline and follow-up points

## Abbreviations

BMI: body mass index
CI: confidence intervals
CF: cognitive functioning
ES: effect size
ICC: intra cluster correlation
IQR: inter quartile distance
LDST: letter digit substitution test
LPA: light physical activity
MVPA: moderate-to-vigorous physical activity
OR: odds-ratio
PA: physical activity
RCT: randomized controlled trial
SD: standard deviation
SE: standard error
SSRT: stop-signal reaction time
SST: stop-signal task
TMT: trail making test
VLT: verbal learning test

## Footnotes

1 Cerebrovascular incidents, neuromuscular disorder, cancer, lung disease, arthrosis, rheumatism, heart diseases, severe back pain, osteoporosis, vascular disorder, balance disorder, diabetes, hearing problems, visual impairment, other condition not previously mentioned.

2 Cerebrovascular incidents, cancer, lung disease, arthrosis, rheumatism, heart diseases, severe back pain, osteoporosis, balance disorder, diabetes, hearing problems, visual impairment, other condition not previously mentioned.
